# New-Onset Seizures and Seizure Worsening in the Course of COVID-19 Infection

**DOI:** 10.7759/cureus.63868

**Published:** 2024-07-04

**Authors:** Ekaterina Viteva

**Affiliations:** 1 Neurology, Medical University, Plovdiv, BGR

**Keywords:** medication, epilepsy, eeg, covid-19, seizures

## Abstract

Introduction: The aim of our study is to assess the clinical manifestations, investigation results, and outcomes in Bulgarian patients with seizures in the course of COVID-19 infection.

Methods: We performed an open, prospective study during a 12-month period from January 2021 with the participation of 290 inpatients and outpatients with seizures who attended the Clinic of Neurology at the University Hospital in Plovdiv, Bulgaria. After a detailed anamnesis, they underwent neurological examination, EEG, neuroimaging, and lumbar puncture when needed. There was a prospective one-year follow-up regarding seizure frequency, EEG, and treatment.

Results: In 18 (5.9%) patients, seizures were related to COVID-19 infection. Nine (3.1%) patients had new-onset seizures, and in nine (3.1%) participants with epilepsy, there was a worsening of seizure frequency. New-onset seizures were more likely to occur in people above 65 years of age, within one to two months from the infection diagnosis. In one participant, seizures were related to fever. The most common seizure types were generalized tonic-clonic and focal motor seizures with/without loss of awareness. Antiseizure medications were started in seven participants. Viral encephalopathy was confirmed in two patients, one of them died. EEG showed focal epileptiform activity in four participants. The one-year prospective observation showed a favorable outcome in five patients who were without seizures, had normal EEG, and three were without treatment. Seizure frequency increase or seizure recurrence was typically observed for a short period of time in the epilepsy group. EEG was worsened in one patient and treatment changes were needed in five participants.

Conclusion: In conclusion, our study results provide evidence about the progress and possible relationship between new-onset seizures and seizure worsening with COVID-19 infection.

## Introduction

Seizures have been documented recently as one of the rare neurological presentations of the SARS-CoV-2 virus infection, although their direct or indirect relation to the infection has not been justified in all cases due to insufficient information about the patient’s anamnesis, the lack of a detailed neurological diagnostic workup in the majority of reports, or a follow-up [1,2]. Seizures may be unprovoked, occurring regardless of a causal association in patients with increased seizure susceptibility or a consequence of fever, hypoxia, metabolic derangements, organ failure, or even cerebral damage such as encephalitis or encephalopathy due to invasion of the virus [2-5]. The usual seizure types are generalized or focal onset motor (tonic, clonic, or tonic-clonic) with or without loss of consciousness [1,6,7]. The suggested mechanisms of viral invasion are 1) through the olfactory nerve or other cranial nerves; 2) hematogenous spread into the central nervous system via circulating lymphocytes; 3) a cytokine storm caused by the host immune response resulting in damaged blood-brain barrier and increased leukocyte migration [1,2,8].

In total, only a limited number of cases with new-onset or acute seizures have been reported so far [2,9-17]. Despite the relatively low risk, the possibility of their occurrence in the course of the disease should not be ruled out. The manifestation of seizures in the course of COVID-19 infection may be associated with a variety of diagnostic and therapeutic challenges. Therefore, the conduction of an open, prospective study on most aspects of seizure presentation in Bulgarian patients with this infection, the summary of investigation results, and one-year follow-up will provide additional useful data for the medical practice.

The purpose of our study is to assess the clinical manifestations, investigation results, and outcomes in Bulgarian patients with seizures in the course of SARS-CoV-2 infection.

## Materials and methods

We performed an open, prospective, one-center survey with the participation of 290 inpatients and outpatients with seizures from south Bulgaria. During a 12-month period from January 2021, they attended the Clinic of Neurology at the University Hospital in Plovdiv, Bulgaria for a consultation or hospitalization with the purpose of diagnosis, monitoring, and/or correction of the antiepileptic treatment.

All study procedures were performed after approval of the Local Ethics Commission at the Medical University of Plovdiv, Bulgaria (C-10-4/2020). Every patient was introduced to the study design and signed an informed consent form before participating in all study procedures.

The following inclusion criteria were used: age ≥18 years, patients with seizures, no matter of seizure type, and recent or present COVID-19 infection, confirmed by a rapid antigen test and/or a PCR test. In all participants, the possibility of other causes of seizures, concomitant diseases, medications, poor compliance of patients with epilepsy, and trigger factors, was excluded. The classification of epileptic seizures and epilepsy is in conformity with the ILAE criteria from 2017 [18].

The patients and their relatives were interviewed about seizures, COVID-19 infection, epilepsy and concomitant diseases, medication usage, and trigger factors by a trained neurologist specialized in epilepsy, and a thorough examination of the medical documentation, including seizure diaries, was performed. All participants underwent neurological examination, EEG, neuroimaging (CT scan or MRI), and lumbar puncture when needed. There was a prospective one-year follow-up regarding seizure frequency, EEG, and treatment. Change in seizure frequency was considered clinically important in case it was at least 50% higher or lower.

Variation analysis was used for data processing. The results for quantitative variables were expressed as means ± SD (standard deviation) and the results for qualitative variables as percentages. The principal outcomes were the impact of the infection on seizure frequency/severity, EEG findings, antiseizure medication changes, and adverse events from treatment. They were assessed through patient visits with examination of seizure diaries and EEG investigations.

## Results

In 18 (6.2%) of all 290 participants, the manifestations of seizures were related to SARS-CoV-2 virus infection. We divided them into two groups: 1) nine (3.1%) of them had new-onset seizures as a presenting symptom of a recent or present COVID-19 infection; 2) worsening seizure frequency was reported in nine (3.1%) participants with a diagnosis of epilepsy in the course of COVID-19 infection.

Group 1 had patients with new-onset seizures as a presenting symptom of a recent (within two months from a positive result) or present (within two weeks from a positive result) COVID-19 infection. This group consisted of four men and five women of age between 36 and 86 years (mean age: 64.7±9.9 years). The clinical characteristics of COVID-19 infection and seizures (severity of the COVID-19 infection, time of first seizure after COVID-19 diagnosis, seizure type and number, epilepsy diagnosis, and antiseizure medications) are presented in Table 1. In all patients, seizures were in addition to respiratory symptoms. We accepted the severity of COVID-19 infection as mild when the patient was not hospitalized and recovered completely in a short period of time (within two weeks from infection diagnosis); as moderate when the patient was hospitalized, with pneumonia, but recovered completely; as severe, when the patient was hospitalized and had post-COVID-19 complications. 

**Table 1:** Clinical characteristics of the COVID-19 infection and associated seizures GTCS, generalized onset bilateral tonic-clonic seizures; VPA, valproate; LEV, levetiracetam; CBZ, carbamazepine; ESL, eslicarbazepine, PB, phenobarbital

Patient: age, gender	Severity of COVID-19 infection	Time of first seizure after COVID-19 infection diagnosis	Seizure type	Number of seizures	Epilepsy diagnosis	Antiseizure medication
1. 52 years, male	Moderate	2 weeks	GTCS	2 clusters of 2 seizures	Yes	VPA 1500 mg/d
2.79 years, female	Moderate	2 months	GTCS	1 cluster of 2 seizures	Yes	VPA 1500 mg/d
3.77 years, male	Mild	1 month	GTCS	Epileptic status	Yes	VPA 1500 mg/d
4. 69 years, female	Moderate	20 days	GTCS	4 clusters of 2 seizures	Yes	VPA 1000 mg/d
5.76 years, female	Mild	20 days	Focal motor with dysphasia, without loss of awareness	5/monthly frequency	Yes	LEV 1500 mg/d
6.36 years, male	Mild	20 days	Focal motor with loss of awareness	1 seizure	No	No
7.86 years, female	Moderate	1 month	Focal motor with loss of awareness to bilateral tonic-clonic	4/monthly frequency	Yes	ESL 1200 mg/d
8.39 years, female	Severe	20 days	Focal motor with loss of awareness	2 seizures followed by epileptic status	No	CBZ 800 mg/d, VPA iv, PB im, Diazepam iv
9.66 years, male	Moderate	The infection was diagnosed right after the seizures	GTCS	2 seizures in 2 consecutive days	No	No

The focal neurological symptoms and investigation results (CSF, neuroimaging, and EEG) are shown in Table 2.

**Table 2:** Focal neurological symptoms and investigation results in patients with new-onset seizures associated with COVID-19 infection

Patient	Focal neurological symptoms	CSF	Neuroimaging	EEG
1	No	Normal	CT and MRI - normal	Abnormal focal activity of groups of sharp waves in the right fronto-temporal region with some contralateral propagation (Figure 1)
2	Transient left hemiparesis	Normal	CT and MRI - normal	Slow (theta) background activity and abnormal focal activity of frequent sharp waves and complexes sharp-slow wave in the left fronto-temporal region (Figure 2)
3	No	Normal	MRI - bilateral small hypodense areas (chronic brain vascular disease)	No pathological findings
4	Muscle rigidity for the neck and extremities	Increased protein level - 0.84 g/L, leucocytes - 14.10^6^/L	MRI - bilateral calcifications in globus pallidus	Slow (theta) background activity and abnormal focal activity of high voltage delta waves and complexes sharp-slow wave (encephalopathy) (Figure 3)
5	Right latent hemiparesis, right facial paresis	Normal	MRI - bilateral small hypodense areas (chronic brain vascular disease)	Significant interhemispheric asymmetry – normal activity in the right and low-voltage, disorganized alpha rhythm with groups of sharp waves in the left hemisphere (Figure 4)
6	No	Normal	CT and MRI - normal	No pathological findings
7	No	Normal	CT and MRI - normal	No pathological findings
8	Syndrome of brainstem impairment - anarthria, flaccid quadriplegia, /+/ Babinski sign bilaterally	Increased protein level - 1.39 g/L, leucocytes - 6.10^6^/L, IgG - 220.38 mg/L	CT - normal	Diffuse slow-wave activity (theta and delta)
9	No	Normal	CT - normal	No pathological findings

**Figure 1:**
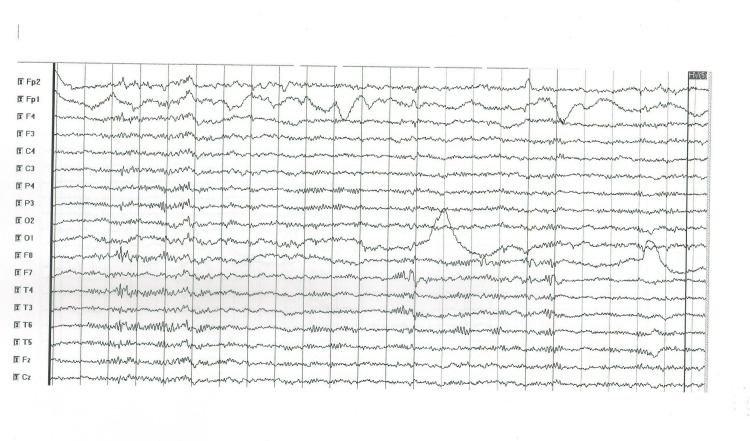
EEG abnormal findings in patient 1

**Figure 2:**
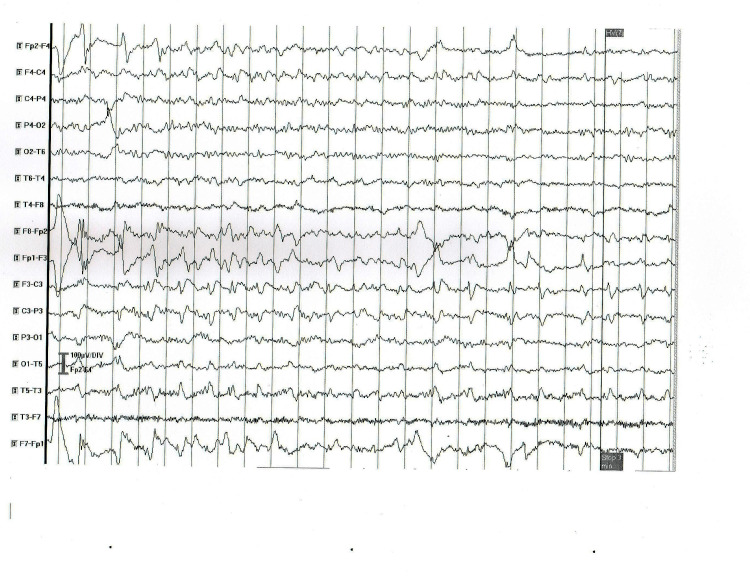
EEG abnormal findings in patient 2

**Figure 3:**
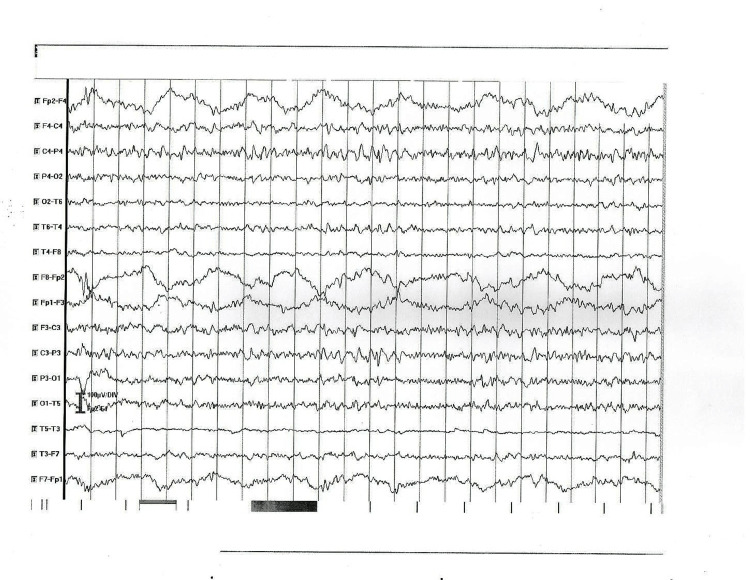
EEG abnormal findings in patient 4

**Figure 4:**
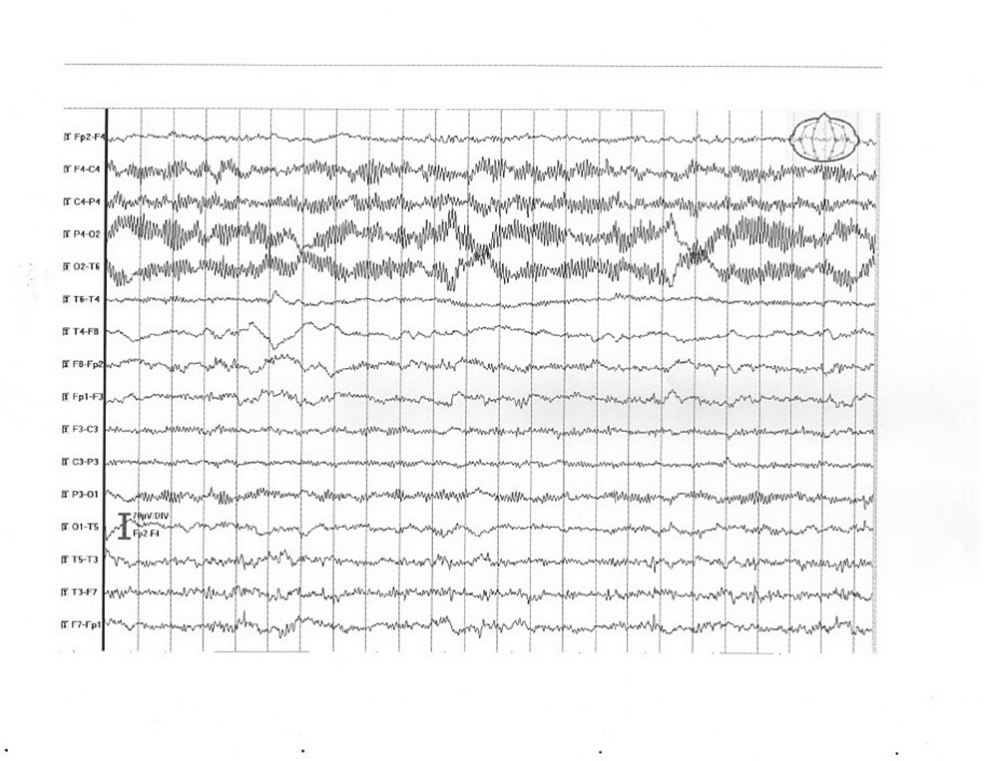
EEG abnormal findings in patient 5

Viral encephalopathy was accepted in patients 4 and 8, based on the clinical course, CSF, and EEG findings. In participants 3 and 5, MRI showed changes consistent with chronic brain vascular disease. The results from the prospective one-year follow-up regarding seizure frequency, EEG, and treatment are included in Table 3.

**Table 3:** Results from the prospective one-year follow-up regarding seizure frequency, EEG, and treatment ESL, eslicarbazepine

Patient	Seizure frequency	EEG	Adverse events from treatment	Antiseizure medications changes
1	No seizures	Normalized	Increased weight	Slow down-titration and termination
2	2 more seizures (1/2 months)	Normalized	No	ESL added
3	1/month	Normal	No	No
4	1/6 months	No change	No	No
5	No seizures	No change	No	No
6	No seizures	Normal	No	No
7	No seizures	Normal	No	No
8	Lethal outcome within 5 days because of progressive brain edema and dislocation	-	-	-
9	No seizures	Normal	-	-

Group 2 had seizure worsening in patients with epilepsy. This group consisted of four men and five women of age between 20 and 62 years (mean age: 41.3±10.6 years). The characteristics of epilepsy are presented in Table 4. 

**Table 4:** Characteristics of epilepsy in patients with seizure worsening associated with COVID-19 infection GTCS, generalized onset bilateral tonic-clonic seizures; VPA, valproate; LEV, levetiracetam; PGB, pregabalin; CBZ, carbamazepine; OCBZ, oxcarbazepine; LCM, lacosamide

Patient: age, gender	Seizure type	Epilepsy type	Recent seizure frequency	Seizure severity	EEG	Treatment
1.43 years, female	Focal with/without loss of awareness, some to generalized tonic-clonic	Refractory focal epilepsy, unknown etiology	1-3/week	Mild	Normal	VPA 1500 mg/d, OCBZ 1500 mg/d, LEV 3000 mg/d
2.35 years, female	Focal with loss of awareness, some to generalized tonic-clonic; GTCS	Refractory, focal and generalized, unknown etiology	1 GTCS/3 months+1 focal/week	Mild+severe	Focal sharp and slow waves in the temporal regions	VPA 1000 mg/d, OCBZ 1800 mg/d, LCM 150 mg/d
3.30 years, female	Complex absences, focal with loss of awareness; generalized tonic	Refractory focal and generalized, structural etiology	10/month	Mild+severe	Focal sharp waves in the bilateral frontal regions	VPA 2500 mg/d, CBZ 800 mg/d
4.62 years, male	GTCS	Refractory, generalized, metabolic (alcohol) etiology	1-2/month	Severe	Normal	VPA 1000 mg/d
5.45 years, male	GTCS	Controlled, generalized, unknown etiology	Without seizures	Severe	Normal	VPA 1000 mg/d
6.37 years, female	GTCS	Controlled, generalized, unknown etiology	1/3 years	Severe	Normal	LEV 2000 mg/d
7.47 years, female	Focal with loss of awareness, GTCS	Refractory, generalized, structural etiology	1/week	Severe	Focal sharp waves and complexes sharp-slow wave in the right fronto-temporal region	VPA 2000 mg/d, LEV 3000 mg/d, PGB 300 mg/d
8.53 years, male	Focal with loss of awareness, GTCS	Refractory, generalized and focal, structural etiology	3-4/month	Severe	Normal	VPA 1500 mg/d, LEV 2000 mg/d
9.20 years, male	GTCS	Controlled, generalized, unknown etiology	Without seizures in the last 5 years	Severe	Focal sharp waves and complexes sharp-slow wave in the right temporal region	VPA 1500 mg/d, CBZ 1200 mg/d

 The severity of COVID-19 infection and its impact on seizures are included in Table 5.

**Table 5:** Severity of COVID-19 infection and its impact on seizures in patients with epilepsy GTCS, generalized onset bilateral tonic-clonic seizures; VPA, valproate; LEV, levetiracetam; BRV, brivaracetam; ESL, eslicarbazepine

Patient	COVID-19 infection severity	Impact on seizure frequency/severity	Impact on EEG	Treatment changes	Prospective 1-year observation results
1	Mild	Increased frequency - daily seizures	No change	No	Previous seizure frequency restored within 1 month
2	Mild	Increased frequency - daily focal seizures	Focal sharp and slow waves in the left temporal region	No	Previous seizure frequency restored within 1 month
3	Moderate	Increased frequency - 20-30 seizures daily	No change	Yes, BRV 200 mg/d added	Previous seizure frequency restored within 1 month
4	Moderate	Increased frequency - weekly seizures	No change	Yes, increased dose of VPA to 2000 mg/d	Previous seizure frequency restored within 2 months
5	Mild	After 20 years without seizures - 3 GTCS within 2 months from infection diagnosis	Focal sharp waves in the fronto-parietal regions	Yes, VPA 1500 mg/d started	No seizures
6	Mild	After 2 years without seizures - 2 GTCS within 1 month from infection diagnosis	No change	No	No seizures
7	Moderate	Increased frequency - 2/week	No change	Yes - ESL added	1 seizure/week
8	Mild	Increased frequency - daily seizures	No change	Yes, increased dose of LEV to 3000 mg/d	Previous seizure frequency restored within 2 months
9	Mild	1 GTCS after 5 years without seizures during the acute stage of infection	No change	No change	1 GTCS in 10 months after the one during the infection - LEV added

The results suggested that seizure worsening in patients with epilepsy during COVID-19 infection was manifested with significantly increased seizure frequency (daily or weekly seizures) or seizure recurrence. It usually occurred within one to two months from the COVID-19 diagnosis, no matter the infection severity, and continued for no more than three months, after which there were no more seizures in two participants, one more seizure in 10 months, or the previous seizure frequency was restored. Seizure worsening was observed in participants with refractory and well-controlled epilepsy and was associated also with seizures in a patient with resolved epilepsy (Patient 5) or without seizures for two or five years (Patients 6 and 9, respectively). EEG worsening was observed in two participants. Treatment changes included a dose increase of some of the antiseizure medications or the addition of a new drug in six patients.

## Discussion

Literature data suggest the neuroinvasive potential of the SARS-COV-2 virus by using the angiotensin-converting enzyme-2 receptor to infect human cells [13]. The frequency of new-onset seizures and seizure worsening in the course of infection varied from 0.4% to 11.2% in clinical investigations [14,15]. It was 5.9% in our study participants. The results from our study confirmed the possible link between new-onset seizures and recent or present COVID-19 infection and seizure worsening in patients with epilepsy in the course of COVID-19 infection. Multiple studies reported a variety of characteristics: 1. Clinical manifestations of new-onset seizures - focal or generalized onset, clusters of seizures, and status epilepticus in the course of COVID-19 infection; 2. Age of patients - children or older people; 3. EEG findings - normal, focal epileptiform discharges, or consistent with status epilepticus; 4. CSF - results - normal or abnormal; 5. Antiseizure medications - type, monotherapy, or combination therapy; 6. Outcome - favorable, severe disability, or death, especially in case of severe COVID-19 infection. Most of them were single case studies, case series, or retrospective studies [2,9,10,12-17,19]. Many patients in these studies had comorbidities or specific neurological problems and received medications [2,15,20]. Therefore, the etiology of seizures was more likely not related to COVID-19 infection only. Besides, the examination of participants was not thorough in many cases; EEG exams, CSF analysis, and/or brain imaging studies were not performed; and there was no follow-up regarding seizure control, EEG, and medications [2,14,20-29].

According to our results, new-onset seizures as a presenting symptom of a recent COVID-19 infection were more likely to occur in people above 65 years of age, within one to two months from the infection diagnosis, no matter its severity. Seizures could be associated with fever during the acute stage of infection (patient 9) or occur during the subacute stage of infection. The most common seizure types were generalized tonic-clonic and focal motor with/without loss of awareness that might occur as single or more typically as seizure clusters and even epileptic status. Epilepsy diagnosis was not confirmed in all cases, and antiseizure medications were not needed in all participants. The clinical manifestations and investigation results confirmed viral encephalopathy as the cause of seizures (in patients 4 and 8, one of them unfortunately died). EEG visualized focal epileptiform activity in four participants (patients 1, 2, 4, and 5). The one-year prospective observation of eight participants was very useful. It showed a favorable outcome in five patients who were without seizures, had normal EEG, three were without treatment, and in one patient, valproate was down-titrated and terminated. The results from a study by Fernandez et al., 2022, were similar in most aspects to our study results. Of 232 cases with confirmed COVID-19, 26 (11.2%) presented with seizures, most often during the first week of infection. Eight (30.7%) participants had GTCS (generalized onset bilateral tonic-clonic seizures), eight (30.7%) had focal seizures with impaired awareness, seven (26.9%) had status epilepticus, and four (11,7%) had secondarily generalized seizures. Other neurological symptoms were found in six (23.1%). Seizure predisposing factors and encephalopathy were confirmed in some participants. Regarding investigation results, CSF was abnormal in four patients, CT was normal in 11 patients, and with chronic vascular disease in five patients. EEG was abnormal with slowing with or without focal epileptiform activity. Antiseizure medications were used in 22 (84%); of them, 17 were on monotherapy (levetiracetam (LEV) or lacosamide (LCM)) and five were on combination therapy. The outcome was favorable in 75%, but five patients died due to pneumonia or status epilepticus, and two patients were with partial recovery [14].

Our data suggested that there was a risk of seizure worsening in patients with epilepsy during COVID-19 infection. Seizure frequency increase or seizure recurrence for a short period of time (within one to two months from infection diagnosis) was typically observed in the acute and subacute stages of the COVID-19 infection of various severity. EEG worsening was found in some patients and treatment changes were made when needed. Seizure worsening was also reported in the literature so far, but in most studies, it was explained by anxiety, depression of patients, worse medication supply, disruption of availability, and quality of care; factors that were excluded in our participants [14]. In some patients with epilepsy, seizures were the initial presentation of COVID-19 infection [12,20,22,29].

Our observational study has some limitations. The sample size was relatively small, although the presented results were useful in showing the main aspects of clinical manifestations and clinical course in patients with new-onset seizures or seizure worsening during COVID-19 infection.

## Conclusions

In conclusion, our study results provide evidence about the possible relationship between new-onset seizure occurrence and seizure worsening in patients with epilepsy in the course of COVID-19 infection. We have summarized the variety of clinical manifestations, trigger factors, EEG, CSF, and neuroimaging findings, as well as the clinical course in patients with new-onset seizures and COVID-19 infection. In patients with epilepsy, seizure recurrence or seizure frequency increase was not associated with epilepsy type or infection severity, although it necessitated treatment optimization in some cases for recovery achievement. Despite the useful information we have provided from our observational study, additional investigations and follow-up are needed to clarify the cause of seizures and choose the most appropriate treatment.
